# Plasma PD-L1 as a biomarker in the clinical management of glioblastoma multiforme—a retrospective cohort study

**DOI:** 10.3389/fimmu.2023.1202098

**Published:** 2023-07-17

**Authors:** Aetsam Bin Masood, Sajida Batool, Sajid Nazir Bhatti, Asad Ali, Marian Valko, Klaudia Jomova, Kamil Kuca

**Affiliations:** ^1^ Department of Biosciences, COMSATS University Islamabad, Islamabad, Pakistan; ^2^ Neurosurgery Department, Pakistan Institute of Medical Sciences (PIMS), Islamabad, Pakistan; ^3^ Department of Medical Lab Technology, Muslim Youth University, Islamabad, Pakistan; ^4^ Faculty of Chemical and Food Technology, Slovak University of Technology in Bratislava, Bratislava, Slovakia; ^5^ Department of Chemistry, Faculty of Natural Sciences and Informatics, Constantine The Philosopher University in Nitra, Nitra, Slovakia; ^6^ Faculty of Science, Department of Chemistry, University of Hradec Kralove, Hradec Kralove, Slovakia; ^7^ Biomedical Research Center, University Hospital Hradec Kralove, Hradec Kralove, Czechia

**Keywords:** GBM, PD-L1, blockade therapy, immune checkpoint inhibitors, liquid biopsy, PD-L1

## Abstract

**Background and objectives:**

Glioblastoma multiforme (GBM) is the most aggressive, malignant, and therapy-resistant tumor of the brain. Blockade therapy targeting the programmed cell death protein 1 (PD-1)/programmed death ligand (PD-L1) axis is currently under investigation for the clinical management of the GBM. This study has quantified the plasma levels of PD-L1 as a biomarker for the clinical management of GBM.

**Methods:**

A cohort (*n* = 128) of Pakistani adult glioblastoma patients together with age- and sex-matched healthy controls was used for quantification of pre-surgery levels of plasma PD-L1. PD-L1 protein and mRNA were measured by PD-L1 platinum enzyme-linked immunosorbent assay and quantitative real-time PCR, respectively. Receiver operating characteristic (ROC) curve analysis was used to compute area under the curve (AUC) for specificity and sensitivity analyses. The Kaplan–Meier survival analysis was employed to compute overall survival.

**Results:**

PD-L1 protein and mRNA were significantly higher in GBM compared to the healthy controls (*p* < 0.0001). Mean PD-L1 concentration for the GBM was found to be 48.98 ± 2.290 pg/ml compared to 27.63 ± 1.281 pg/ml for controls. Gene expression analysis showed statistically significant upregulation (*p* < 0.0001) of PD-L1 in blood of GBM compared to healthy controls. Plasma PD-L1 showed an AUC of 0.840 (*p* < 0.0001; 95% CI = 0.7716 to 0.9090) where a cutoff value higher than 46 pg/ml demonstrated 100% specificity and 57.81% sensitivity. Higher pre-surgery levels of PD-L1 were found to be associated with overall poor survival [*p* < 0.0001; HR (log-rank) = 0.08; 95% CI = 0.04 to 0.15]. Age, gender, and ethnic background were not found to be associated with plasma PD-L1 levels.

**Conclusion:**

The study concludes that blood-based measurements of PD-L1 in GBM can be a promising prognostic marker and therapeutic target besides a rapid and relatively non-invasive screening tool for routine clinical management. Future work extending the analysis to larger cohorts through multi-center collaborations involving pre-treatment and post-treatment groups is required to fully explore the usefulness of circulating PD-L1 for effective clinical applications.

## 1 Introduction

Glioblastoma multiforme (GBM) is a highly aggressive, malignant, and therapy-resistant primary brain tumor with poor prognosis. GBM is also the most abundant of malignant brain tumors accounting for nearly 50% of reported gliomas in USA alone (1). Though much has been learned over the past decades about the progression of GBM, the prognosis, however, remains poor. Studies have reported the median survival time of less than 24 months with a majority of afflicted succumbing to death within 12 months of the diagnosis (2). Magnetic resonance imaging (MRI) remains the gold standard for the diagnosis of GBM, yet the disease is already at an advanced stage by the time imaging reveals a tumor lesion in the brain (3). Surgical resurrection of the tumor mass is the standard first step towards therapeutic control followed by adjuvant therapy, making combined use of radiation and chemotherapy. Despite the multimodal treatment regimen, recurrence and relapse are almost always inevitable; hence, treatment of GBM remains a challenge for clinicians and biologists despite their best efforts (4).

Immune checkpoint inhibitors have been reported to confer anti-tumor resistance by augmenting the ability of immune system to eliminate the cancerous cells (5). Lately, the PD-1/PD-L1 pathway has become of interest for the clinical management of multiple cancers (6). Immune checkpoints are the inhibitory pathways regulating the cellular and systemic immune cascades through ligand–receptor interactions. These checkpoint mechanisms ensure the tight balance between protective and damaging impacts of the immune pathways and can therefore be exploited using specific antibodies for anti-tumor immunity (7). Programmed cell death protein 1, also known as PD-1, is one such transmembrane surface protein encoded by the CD274 gene located on chromosome 9. The main ligand for PD-1 protein is programmed death ligand (PD-L1), also known as B7 Homolog 1 (B7H1), which induces a co-inhibitory signal for the activated T cells expressing PD-L1. This B7H1/PD-L1 pathway inhibits the activity of cytotoxic T cells promoting their apoptosis and functional exhaustion and, hence, facilitating immune evasion of the tumor cells (8). Thus, immunotherapy by blocking these immune checkpoint inhibitors has been attempted and found promising for the treatment of many solid tumors (9). Monoclonal antibodies against the PD1/PD-L1 axis are now among the prescribed cancer treatment regimen (7).

PD-L1 has been shown to be upregulated in GBM and is particularly associated with poor prognosis, malignancy, and aggressiveness (10). Extracellular vesicles that promote the progression of GBM are also shown to express PD-L1 on their surface and therefore are considered to participate in immunosuppression (11). These findings have prompted the evaluation of immune checkpoint inhibitors for the management of hitherto unmanaged GBM (12). Many phase II and phase III clinical trials are now underway to develop antibodies against PD-L1/PD-1 nexus and to investigate new immune checkpoint inhibitors in an effort to therapeutically manage the devastating GBM (13). Besides being used as a therapeutic target for anti-tumor therapy, the levels of PD-L1 in the tumor microenvironment have also been shown as promising prognostic markers (10, 14). However, invasive procedures such as surgical excision and biopsy are required to extract enough tumor tissue material for the measurements of the levels of PD-L1, somewhat constraining the usefulness of this assay in clinical settings (15).

A soluble form of plasma PD-L1 thought to originate from PD-L1-expressing cells in the tumor microenvironment has attracted notable attention as a surrogate marker (16). Many research studies have reported the use of plasma PD-L1 as a reliable prognostic marker in multiple cancers and particularly for the evaluation of the efficacy of the checkpoint blockade therapies (17). Blood-based biomarkers have always been of clinical interest owing to their relatively non-invasive, painless, and cost-effective nature. The present study endeavored to measure the levels of plasma PD-L1 in the blood of pre-operative GBM patients in an effort to document their role in cancer prognosis and clinical management. To our knowledge, this is the first report documenting the levels of plasma PD-L1 from a local cohort of Pakistani origin. The findings of this work are expected to contribute to a broader understanding of the sensitivity, specificity, and reliability of plasma PD-L1 as a biomarker in the GBM tumor management.

## 2 Methods

### 2.1 Collection of blood samples

Adult patients with clinically confirmed diagnosis of glioblastoma (GBM) were recruited to participate in the study from the Neurosurgery ward of the Pakistan Institute of Medical Sciences (PIMS), Islamabad after granting ethical approval from both the ethical review board (ERB) of COMSATS University Islamabad (CUI) and PIMS hospital. A written informed consent was obtained from all the study participants wherein each was informed about the research prior to collecting the blood. Healthy controls were selected with no prior history of malignant disease, diabetes, cardiovascular disease, and/or any other major illness. Controls were matched for age and gender with the malignant cohort. All the study subjects in the GBM cohort were followed up for 6 months after initial surgical excision of the tumor.

To determine the efficacy and usefulness of plasma PD-L1 as a biomarker in the therapeutic management of GBM, blood samples were collected from 64 confirmed GBM patients prior to their initial surgery. Venous blood (3 ml) was drawn by the trained hospital phlebotomist in a pre-labeled ethylene diamine tetra-acetic acid (EDTA) tube to prevent clotting following standard clinical practices. A questionnaire about the disease-related information was also completed by each participant of the study for demographic analysis. The tubes were then safely transferred to the laboratory at the Department of Bioscience, CUI, in an ice box and plasma was separated. First, the blood samples were centrifuged at low speed to separate the aqueous phase and then at high speed to remove residual cells, and finally, the separated plasma was stored at −20°C until further use.

### 2.2 Extraction of RNA and DNase treatment

RNA from the pelleted cells obtained after centrifugation, described in the previous step, was extracted using TRIzol reagent (Thermo Scientific) following their protocol. RNA was dissolved in RNase-free water and was quantified using NanoDrop spectrophotometer and thereafter subjected to DNase (Thermo Scientific) treatment to remove contaminating genomic DNA. First, the DNase enzyme was added to the RNA solution and incubated for an hour at 37°C as described in the manufacturer’s assay. After incubation, samples were re-extracted using phenol-chloroform-isoamyl alcohol (PCI, Invitrogen), and finally, the RNase-free water was added to dissolve the RNA and stored at −20°C.

### 2.3 cDNA synthesis and quantitative real-time PCR

RNA was reverse transcribed using the first-strand cDNA synthesis kit (Thermo Scientific) as per their protocol. Reaction mix consisted of RNase inhibitor, reaction buffer, and dNTPs together with reverse transcriptase (RT) enzyme. After the completion of the reaction, cDNA was stored at −20°C until further analysis. qRT-PCR (Applied Biosystems) was performed using maxima SYBR^®^ green master mix (Thermo Scientific) with β-actin gene as internal control and gene-specific PD-L1 primers.

### 2.4 ELISA for measurement of plasma PD-L1

PD-L1 in the plasma was measured using a commercially available assay (Human PD-L1 Platinum ELISA Kit BMS2212, Thermo Fisher Scientific) according to the protocol. Briefly, wash buffer and assay buffers were prepared and human PD-L1 standard was reconstituted and dilutions were prepared. Samples and controls were added to the micro-well plate and kit-supplied biotin conjugate was then added to each of the well. This was followed by the addition of Streptavidin-horseradish peroxidase and tetramethylbenzidine (TMB) substrate solution. The reaction was terminated by the addition of the stop solution and optical density (OD) values were measured using the automated microplate reader (AMP) Platos R II at a primary wavelength of 450 nm with 620 nm as the reference wavelength. All the measurements were made in triplicate.

### 2.5 Statistical analysis

The differences between two groups were analyzed by Student’s *t*-test or Mann–Whitney *U*-test based on the normality of the data. The chi-square test was used to compare categorical variables between different study groups. One-way analysis of variance (ANOVA) was used to compare more than two groups. Pearson’s or Spearman’s correlation was used to assess associations among different parameters as appropriate. Kaplan–Meier’s survival analysis was carried out to compute overall survival subsequent to initial surgery. The specificity and sensitivity of PD-L1 measurement as biomarker were determined by the area under the curve (AUC) in receiver operator characteristic (ROC) curves. Microsoft Excel (Microsoft Corporation) and GraphPad Prism V9.0 (GraphPad Software, San Diego, CA) were used for all the statistical analyses.

### 2.6 Role of the funding bodies

The funding bodies did not play any role in the study design, data collection, data analysis and interpretation of the data, and writing of this manuscript. The corresponding authors were responsible for undertaking the study and ensuring the accuracy and integrity of the work. All authors have read and approved the final version.

## 3 Results

### 3.1 Demographic characteristics of the study cohort

The study cohort consisted of 128 individuals, of whom 64 were confirmed GBM patients and 64 were age- and sex-matched healthy controls. **Table 1** summarizes the demographic profile of the study participants. Majority of the GBM patients were in the age group of 20–39 years and showed varied ethnic backgrounds. All the subjects belonging to the GBM cohort were asked about the family history of brain tumors, and it was observed that 7.81% of them were affirmative while 92.18% did not have familial past.

**Table 1:** Summary of the demographic features of the study cohort.

Demographic parameters	Controls (*n* = 64)	GBM (*n* = 64)	*p*-value^*^
**Gender**			>0.99
Male	34 (53.12%)	34 (53.12%)	
Female	30 (46.87%)	30 (46.87%)	
**Age (years)**			0.89
Mean age	37.70	37.94	
10–19	2 (3.12%)	2 (3.12%)	
20–29	24 (37.50%)	23 (35.93%)	
30–39	25 (39.06%)	23 (35.93%)	
40–49	6 (9.37%)	7 (10.93%)	
50–59	5 (7.81%)	6 (9.37%)	
>60	2 (3.12%)	3 (4.68%)	
**Ethnicity**			0.12
Punjabi	41 (64.06%)	37 (57.81%)	
Seraiki	0	1 (1.56%)	
Kashmiri	2 (3.12%)	5 (7.81%)	
Pushtun	15 (23.43%)	20 (31.25%)	
Gilgiti	6 (9.37%)	1 (1.56%)	
Family History			
Yes	–	5 (7.81%)	
No	–	59 (92.18%)	

^*^Chi-square test was used to compare categorical variables and t-test was used for quantitative variables.

### 3.2 Plasma PD-L1 levels are higher in GBM

**Figure 1:**
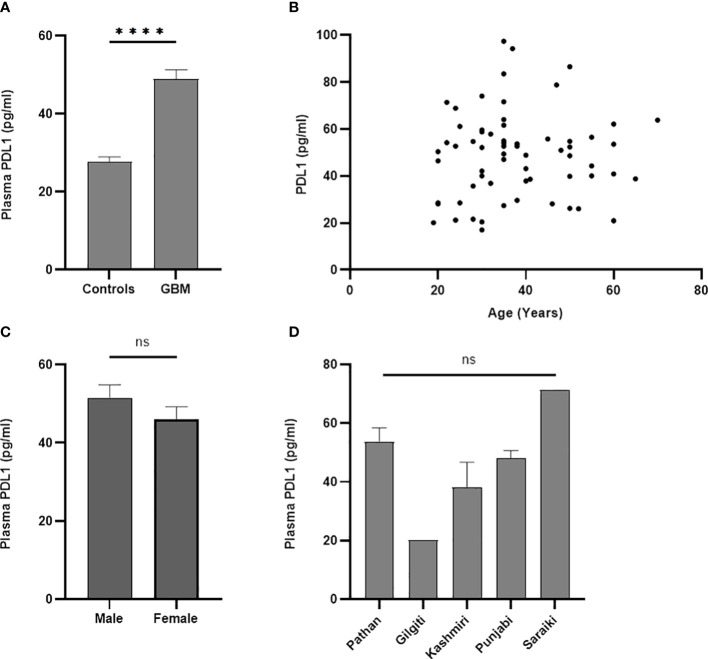
Comparative analysis of concentration of plasma PD-L1 between controls and GBM. A statistically significant difference (*p* < 0.0001) in the concentration of plasma PD-L1 was found between controls and GBM **(A)**. Plasma PD-L1 levels were found to have no association with age (*p* = 0.64) **(B)**, gender (*p* = 0.23) **(C)**, and ethnicity (*p* = 0.12) **(D)**. The '*' sign is used to represent the statistical significance. The 'ns' refers to 'not significant'.

PD-L1 concentration was found to be statistically significantly different between controls and GBM (*p* < 0.0001) as shown in **Figure 1A**. Mean plasma concentration of PD-L1 was 27.63 ± 1.281 pg/ml for controls and 48.98 ± 2.290 pg/ml for GBM. These concentrations were calculated using a standard curve (*R* ^2 = ^0.9971) where the concentrations of known standards ranged from 9.4 pg/ml to 300 pg/ml. Pearson correlation found no statistically significant association between plasma PD-L1 levels and age (*p* = 0.6445, *r* = 0.058, 95% CI = −0.1898 to 0.3003) as can be seen in **Figure 1B**. Male patients were found to have mean plasma PD-L1 concentrations of 51.56 ± 3.272 pg/ml and female patients had 46.05 ± 3.152 pg/ml (**Figure 1C**) with no statistically significant difference (*p* = 0.2330). One-way ANOVA found no significant differences in plasma PD-L1 among different ethnic groups (**Figure 1D**) (*p* = 0.123; *f* = 1.86; *R* ^2 = ^0.113).

### 3.3 PD-L1 gene expression was upregulated in the GBM cohort

**Figure 2:**
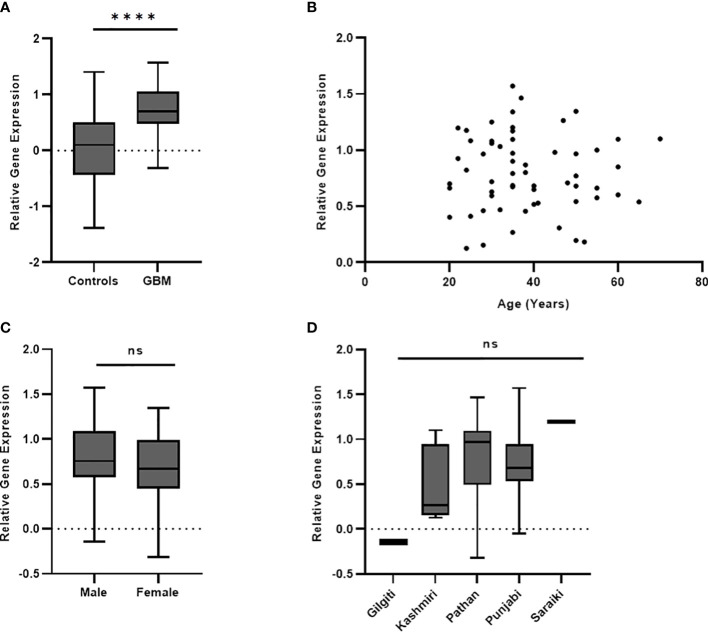
PD-L1 gene expression in blood was significantly different (*p* < 0.0001) between controls and GBM **(A)**. PD-L1 gene expression was not found to have association with age (*p* = 0.64) **(B)**, gender (*p* = 0.21) **(C)**, and ethnicity (*p* = 0.05) **(D)** of study participants. The '*' sign is used to represent the statistical significance. The 'ns' refers to 'not significant'.

PD-L1 was significantly upregulated in peripheral blood of GBM compared to the healthy controls (*p* < 0.0001) as shown in **Figure 2A**. Correlation analysis showed no statistically significant association (**Figure 2B**) of gene expression with age (*p* = 0.6463; *r* = 0.058; 95% CI = −0.1901 to 0.3000). The gene expression was not found to be significantly different (**Figure 2C**) between male and female patients (*p* = 0.2121). Ethnicity of the study participants was also not found to be associated with PD-L1 gene expression (*p* = 0.051; *f* = 2.511; *R* ^2 = ^0.1455) as can be seen in **Figure 2D**.

### 3.4 PD-L1 sensitivity and specificity as biomarker

**Figure 3:**
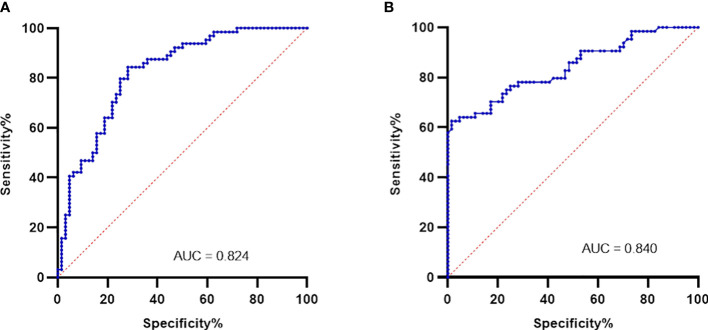
Plasma PD-L1 levels as biomarkers were evaluated using ROC analysis. PD-L1 gene expression in blood gave an AUC of 0.824 (*p* < 0.0001) as shown in **(A)**. Plasma levels of PD-L1 protein was found to have an AUC of 0.840 (*p* < 0.0001) and is shown in **(B)**.

ROC curve analysis was undertaken to determine the specificity and sensitivity of the plasma levels of PD-L1 gene and protein. AUC of ROC of PD-L1 gene expression in blood (**Figure 3A**) was found to be 0.8245 (*p* < 0.0001; 95% CI = 0.7529 to 0.8960). AUC of ROC calculated from the measured values of circulating PD-L1 (**Figure 3B**) was observed to be 0.840 (*p* < 0.0001; 95% CI = 0.7716 to 0.9090). Based on Youden index, a cutoff value of less than 46 pg/ml showed a specificity of 98.44% and a sensitivity of 59.38%. A value higher than 46 pg/ml demonstrated 100% specificity and 57.81% sensitivity. **Table 2** summarizes the demographics of GBM cohort based on the low (<46pg/ml) and high (>46pg/ml) plasma PD-L1.

**Table 2:** Distribution of the GBM cohort based on the low (≤46 pg/ml) and high (≥46 pg/ml) plasma PD-L1.

Demographic parameters	Plasma PD-L1 (≤46 pg/ml)	Plasma PD-L1 (≥46 pg/ml)	*p*-value^*^
No. of patients	26 (40.62%)	38 (59.37%)	
Mean PD-L1 (pg/ml)	31.63	60.85	<0.0001
Gender			0.35
Male	12 (46.15%)	22 (57.89%)	
Female	14 (53.84%)	16 (42.10%)	
Mean age (years)	41.69	35.37	0.04
Mean survival time (months)	6	5	<0.0001

^*^Chi-square test was used to compare categorical variables, t-test was used for quantitative variables, and survival was compared by Kaplan–Meier analysis.

### 3.5 Kaplan–Meier survival analysis found an overall poor survival

**Figure 4:**
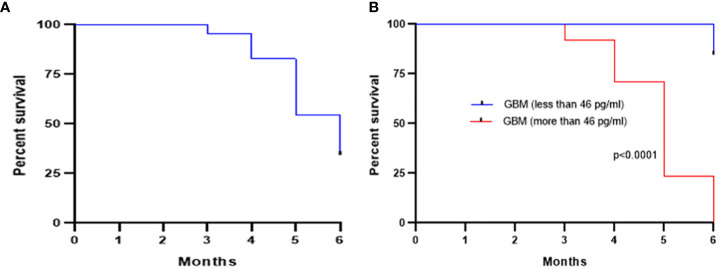
Kaplan–Meier survival curve showing an overall survival of 34.37% after 6 months of initial surgery based on the levels of plasma PD-L1 **(A)**. Further stratification of the GBM cohort based on a cutoff of 46 pg/ml (calculated from ROC curve) showed a statistically significant (*p* < 0.0001) improved survival for low pre-surgery levels of plasma PD-L1 **(B)**.

The GBM cohort was followed for 6 months after initial surgery and survival was found to be 34.37%, as shown by the Kaplan–Meier curve in **Figure 4A**. The GBM cohort was classified into two groups based on the Youden index cutoff value of 46 pg/ml, and a statistically significant difference [*p* < 0.0001; HR (log-rank) = 0.08; 95% CI = 0.04 to 0.15] between these two groups was observed (**Figure 4B**).

## 4 Discussion

GBM is categorized as grade IV glioma with an overall poor survival and is still a challenge to be managed by conventional therapies, i.e., surgical removal, chemotherapy, and radiation (12). The tumor microenvironment created by GBM is conducive for immune evasion and suppression of the normal immune curbing of the cancer spread. Immune checkpoint inhibitors therefore offer a promising avenue for the control of such difficult-to-manage and therapy-resistant tumors (18). The PD-1/PD-L1 pathway is one such immune regulatory nexus that has been shown to have a promising anti-tumor role in the non-small cell lung cancer, head and neck squamous cell carcinoma, cervical cancer, renal cancer, gastric cancer, chronic Hodgkin’s lymphoma, hepatocellular carcinoma, urothelial cancer, and melanoma (12). Though the efficacy of the PD-1/PD-L1 blockade therapy is yet somewhat controversial in the clinical management of GBM, it still has shown an overall reduction in the tumor burden combined with the activation of local and systemic immune responses suppressed by the tumor microenvironment (19, 20). The amount of PD-L1 protein is critical in predicting the therapeutic efficacy of PD-1/PD-L1 blockade therapy in different types of cancers (21). It is therefore important to investigate the expression of PD-L1 in GBM to comprehensively understand its role in cancer prognosis, diagnosis, and effective therapeutic management.

The present study quantified the levels of RNA and protein of plasma PD-L1 in the blood of the confirmed cases of GBM recruited from local cohort together with age- and sex-matched healthy controls. A large number of study participants were observed to be middle-aged with a mean age of 37.94 ± 1.58 years, a much younger age of onset compared to the other parts of the globe. The median age for most of the primary brain tumors in the developed countries has been reported as 59 years, while for GBM, it was 64 years (22). The incidence of GBM in comparatively young individuals is difficult to explain given the lack of epidemiological studies in our population, though partly this disparity in age could be explained by the fact that Pakistan hosts a larger young population where 64% are under the age of 30 years and overall life expectancy is 65.6 years (23, 24) compared to developed countries where people lived to advanced years. Study participants were asked about their family history of cancer, and it was observed that only approximately 7% were familial cases consistent with the fact that GBM is a sporadic brain tumor (25).

Pakistan is home to many different ethnic sub-groups, and since the study subjects were recruited from one of the largest government-run hospital in the capital city where treatment of GBM is available, all were asked about ethnicities to establish its putative association with PD-L1 expression. It was observed that no correlation existed with the ethnic origin and expression of the PD-L1 RNA and protein. The patients were selected randomly so that bias in sampling can be disregarded for overrepresentation of one ethnic group over the other. One possible reason for Punjabis to be present in larger number among cancer subjects may be that the capital city hosts a large population of Punjabi ethnic group followed by Pushto-speaking Pakhtuns/Pathans. Demographic differences in incidence of GBM have been observed for developed countries, and it was found that GBM was more prevalent in male than in female patients (22, 26).

GBM is currently diagnosed using imaging technologies, particularly magnetic resonance imaging (MRI) with/without contrast, and definitive pathology is confirmed by tissue biopsies. Imaging, though non-invasive, is limited by failing to distinguish actual tumor lesions from treatment-induced lesions and can often lead to diagnostic errors. Tumor biopsy, on the other hand, is highly invasive and can only offer a static glimpse into the tumor microenvironment, which, for GBM, is highly heterogeneous and ever changing with additional risk of hemorrhage and neural dysfunction (27, 28). Liquid biopsies utilizing body fluids particularly blood are minimally invasive and safe alternates for the diagnosis, pre- and post-treatment monitoring, and prognostic predictions in clinical settings. Cerebrospinal fluid (CSF) and peripheral blood are now under intensive investigation for the estimation of biomarkers that can help in the early diagnosis of GBM together with post-treatment management of the disease where tissue biopsy is not possible and/or feasible (29, 30). These minimally invasive liquid biopsies offer an additional advantage for resource-limited settings such as developing countries and underdeveloped societies where healthcare facilities are either non-existent or burdened enough to support the routine imaging and invasive biopsies, which are obvious diagnostic choices but costly.

PD-L1 expression as a putative biomarker in GBM has been investigated with contradictory roles in prognosis mainly within tumors. Plasma PD-L1 has been shown by few other studies as a marker for primary and recurrent brain malignancies with poor prognosis and overall poor survival (17, 31–33). PD-L1 levels have been reported to be higher for other types of aggressive malignancies and are therefore attributed as an independent marker to predict overall survival and treatment efficacy (34, 35). These studies have described the differences in the levels of plasma PD-L1 following radiotherapy in gliomas and anti-PD-1 immune therapy in recurrent glioblastomas as a promising biomarker. However, the measured levels of the plasma PD-L1 were different in all these studies, necessitating further explorative studies involving large and diverse cohorts to determine its clinical usefulness. For effective cancer immunotherapeutic management using blockade therapy, PD-L1 expression in tumor tissues has been approved as companion diagnostics for pembrolizumab-based treatment (36).

Many studies have reported the elevated levels of plasma PD-L1 in several types of malignancies with a potential to be used as a prognostic biomarker but still a baseline has not been established. It therefore is of paramount importance to characterize the expression of plasma PD-L1 in larger cohorts of different ethnicities, races, tumor types, and stages of malignancy for establishing robust and reproducible cutoff values to characterize the progress of the disease and outcome of the therapy. Also, given the role of PD-L1/PD-1 in blockade therapies, the expression has to be characterized to compensate for heterogeneity in the tumor microenvironment and measurement techniques/technologies for the effective applications of immune checkpoint inhibitors. The present study compared the differences in the expression levels of plasma PD-L1 between GBM and healthy controls recruited from local population. Blood was collected from adults with a confirmed diagnosis of GBM prior to their initial surgery, and overall survival was computed following 6 months post-surgery. To our knowledge, this is the first study describing the plasma levels of PD-L1 in a GBM cohort of Pakistani origin. The present study is limited by two important considerations: first, the study has only investigated the pre-surgery levels of plasma PD-L1 and has not contrasted it with post-surgery levels. Second, the number of patients for survival analysis was small because the study recruited participants from the largest tertiary-care hospital in the capital city where treatment to multiple brain malignancies is offered. Hence, the patient pool is highly heterogeneous with clinical presentation of different types of gliomas. This study only focused on GBM, and other gliomas were not the focus of investigation; hence, it reduced the number of eligible participants. For a comprehensive understanding of the role of plasma PD-L1 in overall survival and prognosis of GBM, future studies should compare the pre-surgery and post-surgery groups with a large sample size so that patient groups can be stratified and an optimized therapy regimen be devised.

## Data availability statement

The original contributions presented in the study are included in the article, further inquiries can be directed to the corresponding authors.

## Ethics statement

The studies involving human participants were reviewed and approved by Ethical Committee of PIMS Islamabas, Ethics Review Board of Department of Biosciences, COMSATS University Islamabad. The patients/participants provided their written informed consent to participate in this study.

## Author contributions

Conceptualization: SB and KK. Methodology: AM and AA. Software, AM and SB. Validation: SB, SNB, and KK. Formal analysis: SB, MV, and KJ. Investigation: AM and SB. Resources: SB, MV, KJ, and KK. Data curation: AM, AA, and SB. Writing—original draft preparation: AM. Writing—review and editing: SB, SNB, MV, KJ, and KK. Visualization: SB and AA. Supervision: SB and KK. Project administration: SB and KK. Funding acquisition: MV, KJ, and KK. All authors have read and agreed to the published version of the manuscript.
